# Draft Genome Sequences of Putative Aerobic Anoxygenic Phototrophic Bacterial Strains *Jannaschia* sp. Strains AI_61 and AI_62, Isolated from Seawater around a Coastal Aquaculture Area

**DOI:** 10.1128/MRA.00491-21

**Published:** 2021-07-15

**Authors:** Yuki Sato-Takabe, Yu Nakajima, Satoru Suzuki, Kota Sekiguchi, Satoshi Hanada, Takuhei Shiozaki

**Affiliations:** a Atmosphere and Ocean Research Institute, The University of Tokyo, Kashiwa, Chiba, Japan; b Department of Biological Sciences, Tokyo Metropolitan University, Hachioji, Tokyo, Japan; c Institute for Extra-cutting-edge Science and Technology Avant-garde Research (X-star), Japan Agency for Marine-Earth Science and Technology, Yokosuka, Kanagawa, Japan; d Center for Marine Environmental Studies, Ehime University, Matsuyama, Ehime, Japan; e National Institute of Advanced Industrial Science and Technology, Tsukuba, Ibaraki, Japan; University of Southern California

## Abstract

Here, we report the draft genome sequences of putative aerobic anoxygenic phototrophic bacterial strains *Jannaschia* sp. strains AI_61 and AI_62, isolated from seawater around a coastal aquaculture in Ainan, Ehime, Japan. These genome sequences could be useful for our understanding of the variation of aerobic anoxygenic phototrophs in the genus.

## ANNOUNCEMENT

**FIG 1:**
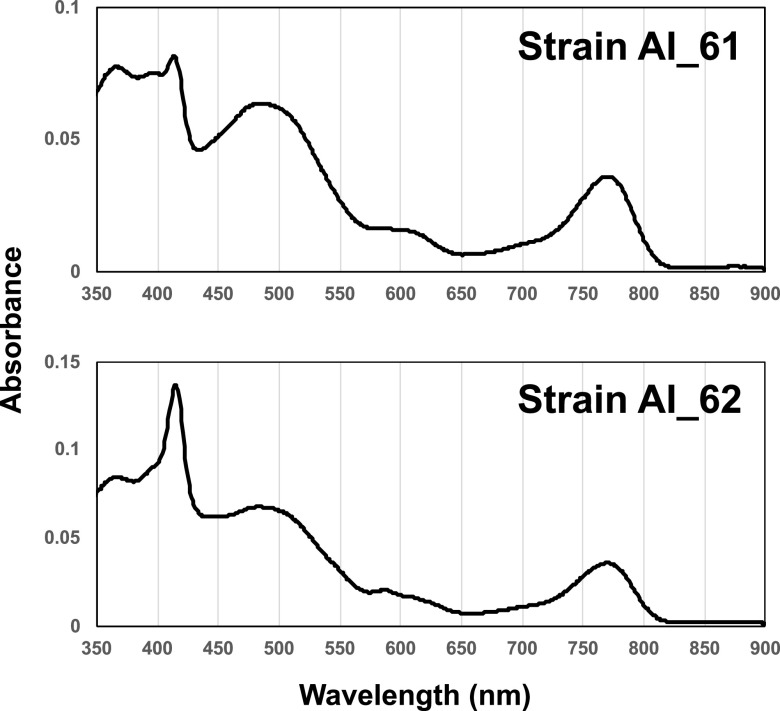
Absorption spectra of strains AI_61 and AI_62.

Aerobic anoxygenic phototrophic bacteria (AAnPB) are photoheterotrophs with a bacteriochlorophyll *a* (BChl *a*) (1). The AAnPB strains *Jannaschia* sp. strains AI_61 and AI_62 are aerobic Gram-negative bacteria belonging to the phylum *Proteobacteria*, which were obtained from seawater at a depth of 1 m around a coastal aquaculture in Ainan, Ehime, Japan (32°56 N, 132°30 E), using buckets. Original seawater was plated directly onto 1/25 diluted ZoBell 2216E agar plates. The plates were incubated at 30°C for 3 days under dark conditions. Single colonies for strains AI_61 and AI_62 were picked up. These two strains produce BChl *a*, indicated by the specific absorbance peak at 770 nm (Fig. 1). Cells of strains AI_61 and AI_62 for *in vitro* absorption spectrum measurement using a UV1800 UV-visible (UV-Vis) spectrophotometer (Shimadzu, Japan) were obtained from culture incubated for 10 days at 30°C on marine agar (BD Difco, USA) in the dark. Cells were disrupted using a sonicator (Ohtake Works, Japan) and extracted by the acetone-methanol (70:30, vol/vol) method. There are 12 currently recognized species in the *Jannaschia* genus, which contains only two BChl *a*-producing species (Jannaschia seohaensis and Jannaschia formosa [2, 3], respectively).

The genomic DNAs of strains *Jannaschia* sp. AI_61 and AI_62, which were grown in ZoBell 2216E liquid medium at 30°C for 14 days under dark conditions, were extracted using the Wizard SV genomic DNA purification system (Promega, Madison, WI). The MGIEasy FS PCR-free DNA library prep set kit (MGI) was used for library preparation, and paired-end sequences (150 bp of each end) were obtained on the DNBseq (MGI) instrument. Genome assembly was performed using SPAdes 3.15.2, with slight modifications (4). The assembled sequences were annotated using the on-line DDBJ Fast Annotation and Submission Tool (DFAST; https://dfast.nig.ac.jp) and Prokka (5), version 1.14.6. Default parameters were used for all software unless otherwise specified. The 16S rRNA gene sequence taxonomic identification using EzBioCloud (6) confirmed that both strains AI_61 and AI_62 are most closely related to Jannaschia seohaensis (96.53% identities). The genome sequences of *Jannaschia* sp. AI_61 and AI_62 consist of 39 and 49 scaffolds, respectively, using SPAdes (total length, 3,963,206 bp and 3,918,448 bp, and *N*_50_, 575,535 bp and 575,536 bp, respectively). The numbers of reads generated for each genome were 7.5 million (2.25 Gbp) and 4.72 million (1.416 Gbp) for AI_61 and AI_62, respectively. The genome sizes were estimated from manual calculation using DFAST. The estimated genome sizes of the strains were smaller than that of the closest relative in the genus *Jannaschia* (4.84 Mbp for *J. seohaensis* DSM 25227 [accession number GCF_900116765.1]). The GC contents (63.3% for AI_61 and 63.3% for AI_62) were relatively lower than that of the closest relative, *J. seohaensis* DSM 25227 (67.8%).

For *Jannaschia* sp. AI_61 and AI_62, DFAST and Prokka identified 3,841 and 3,829 protein-coding sequences (CDSs) for AI_61 and 3,799 and 3,793 CDSs for AI_62, respectively. Both tools also identified 45 tRNA and 3 rRNA genes for both strains.

### Data availability.

The whole-genome shotgun projects of *Jannaschia* sp. AI_61 and AI_62 have been deposited in DDBJ/EMBL/GenBank under the accession number BPFG00000000.1 for the assembly and DRR289363 in DRA11917 for the raw reads and BPFH00000000.1 for the assembly and DRR289364 in DRA11917 for the raw reads, respectively.
